# Rat and human STINGs profile similarly towards anticancer/antiviral compounds

**DOI:** 10.1038/srep18035

**Published:** 2015-12-16

**Authors:** Heng Zhang, Min-Jie Han, Jianli Tao, Zhao-Yang Ye, Xiao-Xia Du, Ming-Jing Deng, Xiao-Yan Zhang, Lan-Fen Li, Zheng-Fan Jiang, Xiao-Dong Su

**Affiliations:** 1 State Key Laboratory of Protein and Plant Gene Research, and Biodynamic Optical Imaging Center (BIOPIC), School of Life Sciences, Peking University, Beijing 100871, China

## Abstract

Cyclic dinucleotides (CDNs) and antitumor/antiviral agents (DMXAA and CMA) trigger STING-dependent innate immunity activation. Accumulative evidences have showed that DMXAA and CMA selectively activate mouse, but not human STING signaling. The mechanism underlying this species selectivity remains poorly understood. In this report, we have shown that human and rat STINGs display more similar signaling profiles toward DMXAA and CMA than that of human and mouse STINGs, suggesting that rat is more suitable for preclinical testing of STING-targeted drugs. We have also determined the crystal structures of both apo rat STING and its complex with cyclic GMP-AMP with 2′5′ and 3′5′ phosphodiester linkage (2′3′-cGAMP), a human endogenous CDN. Structure-guided biochemical analysis also revealed the functional importance of the connecting loop (A140-N152) between membrane and cytosolic domains in STING activation. Taken together, these findings reveal that rat STING is more closely related to human STING in terms of substrate preference, serving as a foundation for the development of STING-targeted drugs.

In innate immune system, cyclic GMP-AMP synthase (cGAS) has been recently identified as a cytosolic double strand DNA (dsDNA) sensor and triggers the interferon induction^1^,^2^. The cGAS engages in the formation of cGAMP through a dsDNA-dependent mechanism^3^,^4^,^5^,^6^. The endogenous cGAMP contains an unusual combination of 2′5′ and 3′5′ phosphodiester linkage (referred to as 2′3′-cGAMP) and binds to and activates the adapter protein STING (stimulator of interferon genes; also known as ERIS, MITA, MPYS and TMEM173)^7^,^8^,^9^,^10^,^11^, thereby initiating a cascade of immune events^1^,^12^. Dimerization of STING was shown to be required for its function and mediated primarily by the N-terminal transmembrane helices^10^. STING also contains a globular C-terminal domain (CTD) connected to the transmembrane helices by a cytoplasmic linker. The STING CTD is responsible for binding to the ligands and recruits kinases such as TBK1 and IKKε, which subsequently phosphorylate and activate the transcription factors IRF3, and induce type I IFN production^7^,^10^,^11^,^13^. In addition to the endogenous 2′3′-cGAMP, other cyclic dinucleotides (CDNs) including chemically synthesized 2′2′- and 3′2′-cGAMP isomers, and bacterial secondary messenger molecules 3′3′-cGAMP, c-di-GMP and c-di-AMP are all capable of activating STING^14^,^15^,^16^.

Structural studies have shown that CDN binding is associated with an open-to-closed conformational change at STING CTD region corresponding to open-inactivated and close-activated states^6^,^17^,^18^,^19^. Chemical agents DMXAA (5,6-Dimethylxanthenone 4-acetic acid) and CMA (10-carboxymethyl-9-acridanone) have been screened and tested for excellent anticancer/antiviral effect earlier in mouse but failed in clinical trials^20^,^21^,^22^,^23^, and their effects have been identified lately to be via induction of type I IFN involved in STING-dependent immune responses^24^,^25^,^26^. Further studies revealed that DMXAA or CMA binds well to mouse STING (mSTING) rather than to human STING (hSTING)^18^,^27^. Although hSTING^S162A^ substitution partly restored the interferon signaling, the underlying molecular mechanisms of the species preference remained unclear^18^.

In the studies presented here, we have discovered that the rat STING (rSTING) has similar profiles both by structural analyses, and by functional assays, and we propose that the rat STING (rSTING) is more appropriate for preclinical evaluation of potential STING-targeted small molecule drugs. Furthermore, extensive biochemical and mutational experiments unambiguously identified certain mutations that are involved in the different STINGs’ preference towards those mouse-specific compounds.

## Results

### *In vivo* and *in vitro* analysis of DMXAA or CMA-induced STING signaling

**Figure 1:**
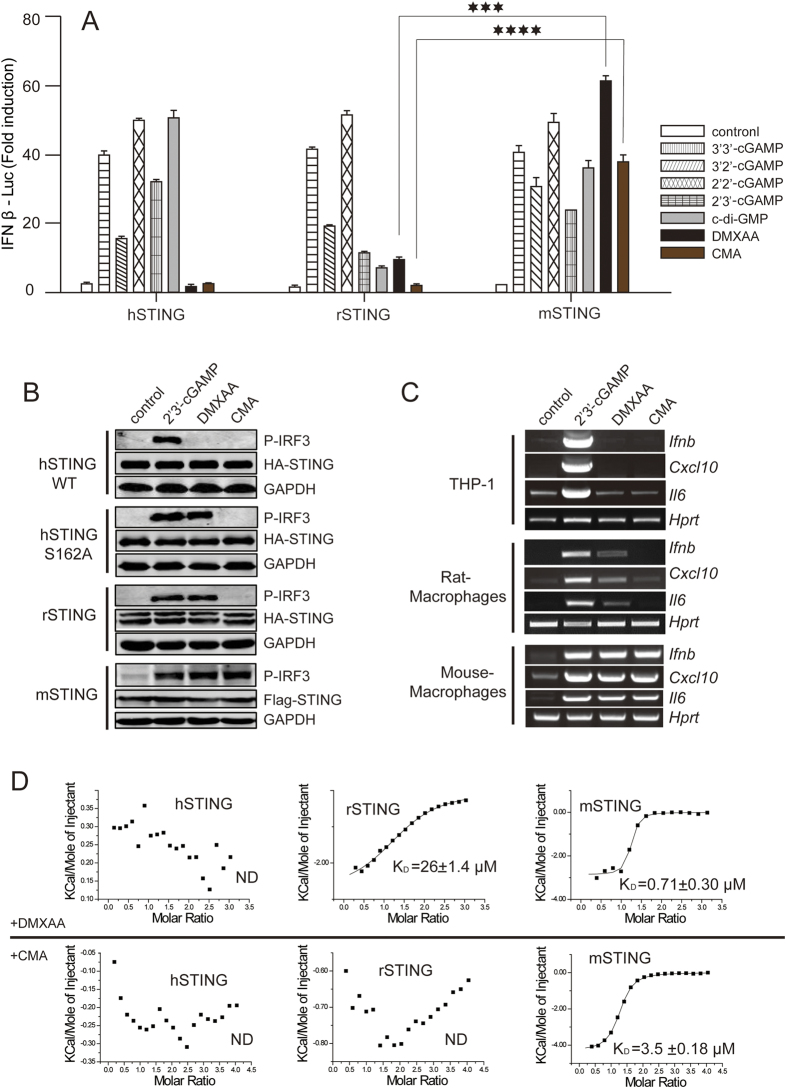
rSTING and hSTING respond in a more similar way to anticancer/antiviral agents DMXAA or CMA than the way of mSTING. (**A**) HEK293T cells were transiently transfected with indicated species of STING plasmids, along with IFN β-Luc reporter plasmids. After 12 hours, cells were PFO-permeabilized to deliver cGAMP linkage isomers (5 μM) or stimulated with DMXAA (266 μM) or CMA (500 μg/mL). Luciferase reporter assay was performed after incubation for an additional 12 hours. Values represent the mean average of triplicate experiments. Error bars indicate SEM. (**B**) Similar to (**A**), 293T cells were transfected with indicated species or mutated STING plasmids and stimulated with 2′3′-cGAMP, DMXAA or CMA. Immunoblot analysis (with anti-p-IRF3, anti-STING and anti-GAPDH) was performed after 12 hours. GAPDH as the loading control. The assays were run under the same experimental conditions and the blots were cropped from original full-sized images (attached as supplemental Fig. S8). (**C**) RT-PCR analysis of indicated STING-pathway related gene expression levels after indicated stimulation in THP-1 (human monocytic cells) or peritoneal macrophages from rat or mouse. HPRT as the loading control. Full RT-PCR are shown in Supplemental Fig. S8. (**D**) ITC binding curves for titration of DMXAA or CMA into indicated species of STING-CTD.

Rat genome shares high sequence similarity with human and mouse, however, both genomic organization and chromosome morphology of rat are closer to that of the human than mouse^28^. In terms of STING sequence homology, rSTING is also between hSTING and mSTING, however, rSTING and hSTING show more similarities on some key residues identified for ligand binding (Fig. s1). Therefore, we first sought to determine if rSTING and hSTING respond in a similar way to anticancer/antiviral compounds than that of mSTING. The IFN-β luciferase assay showed that rSTING and hSTING do not respond or only respond weakly to the anticancer/antiviral drugs, whereas these drugs strongly activated mSTING (Fig. 1a). We also performed Western blot experiments to detect the phosphorylation of endogenous IRF3, a hallmark of STING pathway activation. The results further verified that CMA failed to activate both rSTING and hSTING (Fig. 1b), whereas CMA is a potent activator of mSTING. Moreover, RT-PCR results showed that endogenous mRNA level of *Ifnb*, *Cxcl10* and *Il6* were robustly increased when treated with DMXAA or CMA in murine macrophages, but not in rat macrophages and human THP-1 cells (Fig. 1c). Consistent with the *in vivo* findings, DMXAA or CMA displayed a high binding affinity towards the CTD of mSTING. In contrast, DMXAA or CMA exhibited none or only weak affinity towards hSTING and rSTING (Fig. 1d). These findings demonstrated that rSTING and hSTING respond similarly to DMXAA or CMA.

### Crystal structures of rSTING alone and in complex with cGAMP

**Figure 2:**
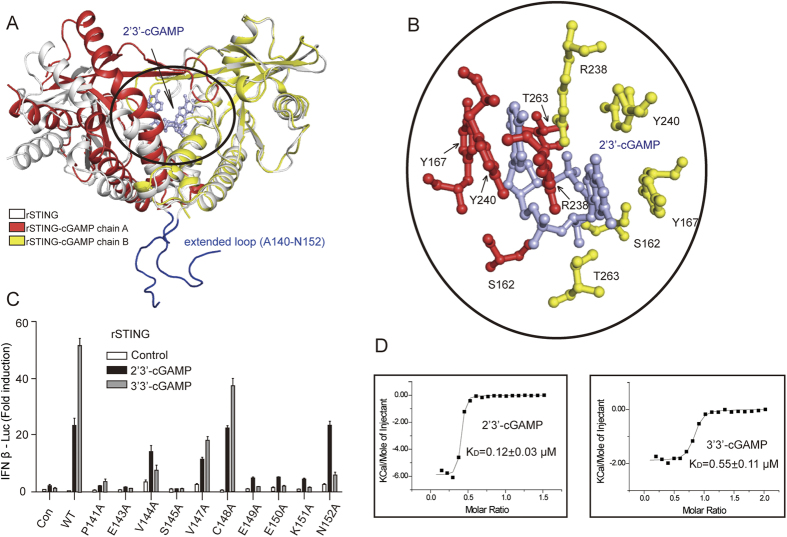
Structural and functional characterization of native rSTING and rSTING -cGAMP. (**A**) Structural alignment of native rSTING and rSTING-cGAMP. The native rSTING (grey) adopts an open conformation compared with the rSTING-cGAMP. The extended loop is shown in light-blue. Each chain of the rSTING-cGAMP is highlighted in a red or yellow color. (**B**) A close-up view of the rSTING-cGAMP interactions. (**C**) Luciferase analysis of the extended loop mutants. HEK293T cells were transfected with indicated rSTING mutations and IFN β-Luc reporter plasmids. A luciferase assay was performed after stimulation with 2′3′-cGAMP or 3′3′-cGAMP. (**D**) The cGAMP binding affinity as measured by ITC.

To elucidate the detailed mechanism of rSTING activation, we have solved the crystal structures of rSTING alone and rSTING in complex with 2′3′-cGAMP (Table 1). To improve the diffraction quality of native rSTING crystals, the high-entropy patch of sequence K338-V341 was substituted for A338-A341^29^, these substitutions have been verified not to affect normal IFN signaling activity of rSTING (Fig. s2). The apo rSTING structure adopts an open conformation, whereas the rSTING-cGAMP complex shows a closed conformation (Fig. 2a). Several native STING structures have been solved, including hSTING, mSTING and two forms of Nematostella vectensis STING (nvSTING)^17^,^30^,^31^,^32^,^33^,^34^. Structural comparison shows that rSTING, hSTING and nvSTING form 1 (rotated “open” form) adopt the open conformation (Fig. s9). However, the closed conformation is observed in mSTING and nvSTING form 2 (unrotated “closed” form). It is possible that the native STING is structural dynamic. Although the overall folding of rSTING is similar to that of hSTING and mSTING, the apo rSTING structure contains an extended loop (A140-N152) that is conserved but absent in any other available STING structures (Fig. 2a, Fig. s1 and Fig. s10), this region was previously predicted to be situated in the membrane, connecting the third and fourth transmembrane regions^7^,^10^,^11^. We performed IFN-β luciferase assays by individually mutating each of the 13 residues in this region, and measured its impact on STING activation. Intriguingly, substitution of these residues with alanine largely impaired the induction of type I IFN, suggesting some important role of this region may play during interferon signaling (Fig. 2c). With the corrected topology of STING after solving crystal structures^17^,^30^,^31^,^32^, this region should be redefined as the connecting region (linker loop) between the soluble C-terminal domain (CTD) and the N-terminal transmembrane domain, it is most likely this linker region in the full length protein contains certain structural elements important to transduce signals between the membrane and cytosol, rather than N-terminal loop as shown in the current crystal structure. Interestingly, all the charged residues in the “linker loop” (E143, E149, E150, and K151) can not be mutated to Ala, suggesting that these residues make interactions with other charged residues of STING. E149R mutation significantly impaired the IFN induction, further demonstrating the importance of the electrostatic interactions (Fig. 2c and s3). Therefore, full length STING structure is needed for a full understanding of the function of the “linker loop”.

In the complex structure, the 2′3′-cGAMP occupies the pocket formed by the dimer and the purine rings stack against Y167 and Y240 by π-π interactions (Fig. 2b). The hydroxyl groups of S162 and T263 and the guanidine group of R238 are hydrogen bonded to 2′3′-cGAMP. Additionally, 2′3′-cGAMP binding is strengthened by hydrophobic and water-bridge interactions, which involve Y163, R232, A239, S241, P264 and T267. Not surprisingly, 2′3′- and 3′3′-cGAMP isomers robustly activated type I IFN production (Fig. 1a), and exhibited high binding affinities for the CTD of rSTING (Fig. 2d).

### Structural features of the lid regions and identification of residue 230 as a key determinant of DMXAA or CMA binding affinity and signaling

**Figure 3:**
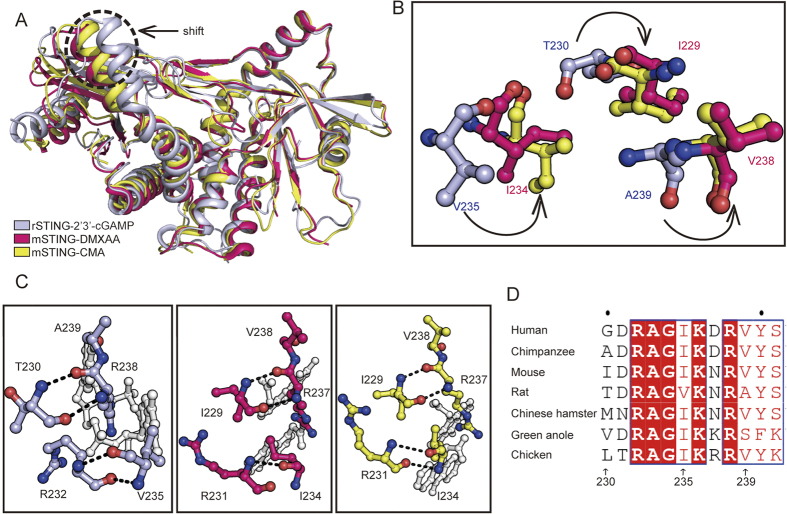
The lid region in rSTING-cGAMP is distinct from that of the mSTING-agents structures. (**A**) Structural comparisons of rSTING-cGAMP (light-blue) with mSTING-DMXAA (red) and mSTING-CMA (yellow). (**B**) A close-up view of the lid region comparison, color scheme is the same as in (a). (**C**) A close-up view of residues 230 and 235 involved in interactions. The color scheme is the same as in (a). The cGAMP or agents is colored grey. The left pane shows rSTING-cGAMP, and the right pane show mSTING-CMA. The middle pane displays the mSTING-DMXAA. (**D**) Sequence alignment of the lid region in STING members. The strictly conserved residues are marked by a red background and the conserved residues are shown in red.

**Figure 4:**
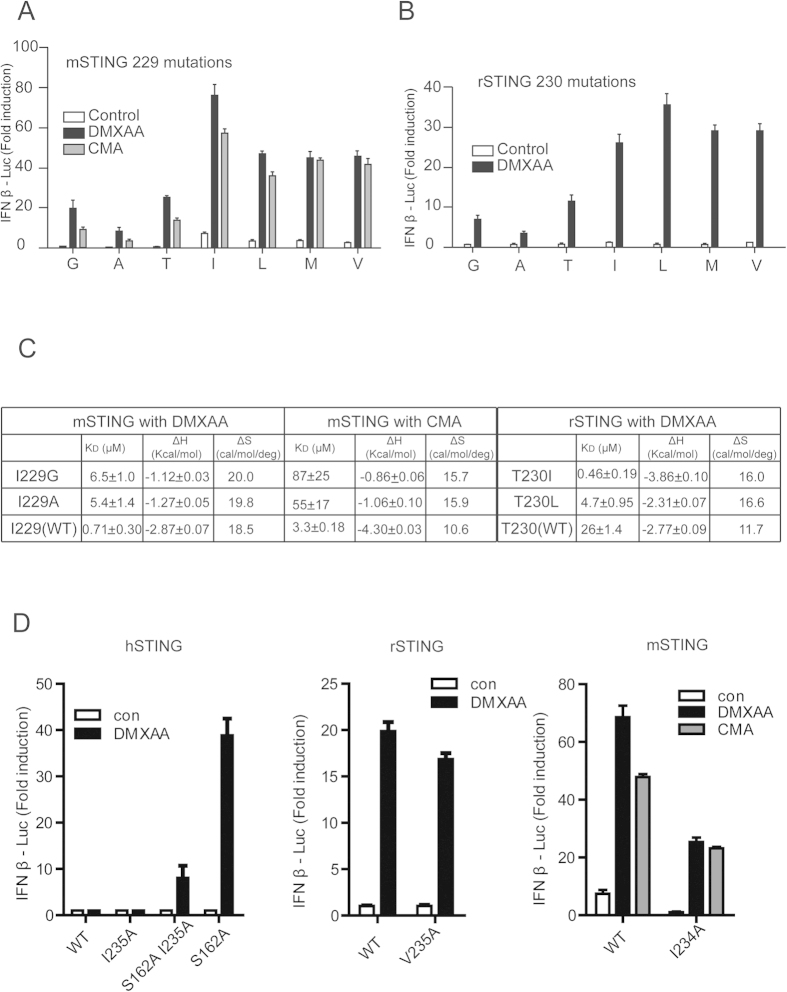
Replacement of residue 230 by bulky hydrophobic amino acid strengthen the agents binding and agents-induced IFN production. (**A**) Similar to Fig.1 (A), Luciferase analysis of the residue 229 mutants in mSTING upon indicated stimulation. (**B**) Luciferase analysis of the residue 230 mutants in rSTING upon stimulated with DMXAA. (**C**) DMXAA or CMA binding studies of STING mutants by ITC. (**D**) Luciferase analysis of the residue 235 mutants in indicated species of STING upon indicated stimulation.

The crystal structure of rSTING-cGAMP is overall similar to that of hSTING- or mSTING-cGAMP, however, subtle and significant structural differences occur between rSTING-cGAMP with mSTING-DMXAA or mSTING-CMA complexes. Binding of cGAMP results in a shift of one chain toward the other chain compared with mSTING-DMXAA or mSTING-CMA to a distance of 5.8 or 7.4 Å, respectively (Fig. 3a). Notably, the lid region is the most different. Binding of DMXAA or CMA triggers a relocation of the lid region, especially position 230 (Fig. 3b), which varies quite much (including glycine, alanine, threonine and hydrophpbic residues) among known STING members (Fig. 3d). We have thus postulated that the residue 230 plays an important role in drug preference. To test this observation, we performed a systematic point mutation scan to change residue 230 to several other typical amino acids. Interestingly, small residue substitutions of mSTING^I229G^, mSTING^I229A^ and mSTING^I229T^ variants (mSTING I229 is equivalent to position 230 in rSTING and hSTING) exhibit dramatically decreased interferon induction in response to DMXAA or CMA (Fig. 4a). Similarly, rSTING^T230G/A^ resulted in a slight decreased interferon production in response to DMXAA. In sharp contrast, a big hydrophobic residue substitution of the position 230/229 (rSTING/mSTING) increased IFN-β production in response to agents (Fig. 4a,b). Consistent with *in vivo* observation, mSTING^I229G^ and mSTING^I229A^ variants resulted in a ~9 and 7-fold reduction in DMXAA binding affinity, respectively (Fig. 4c). For CMA, mSTING^I229G^ and mSTING^I229A^ mutations showed a detectable binding affinity values (K_D_) ranging from 78 to 55 μM, more than 15-fold weaker than that of WT mSTING. Moreover, the DMXAA binding affinity value for rSTING^T230I^ was 0.46 μM, 50-fold greater than that for WT rSTING. Importantly, the hSTING^S162A^ 230 substitutions also displayed similar signaling profiles toward DMXAA (Fig. s4), further validating residue 230 as a critical one for IFN-β induction. Collectively, these results established that residue 230 plays a key role in responding to anticancer/antiviral agents.

**Figure 5:**
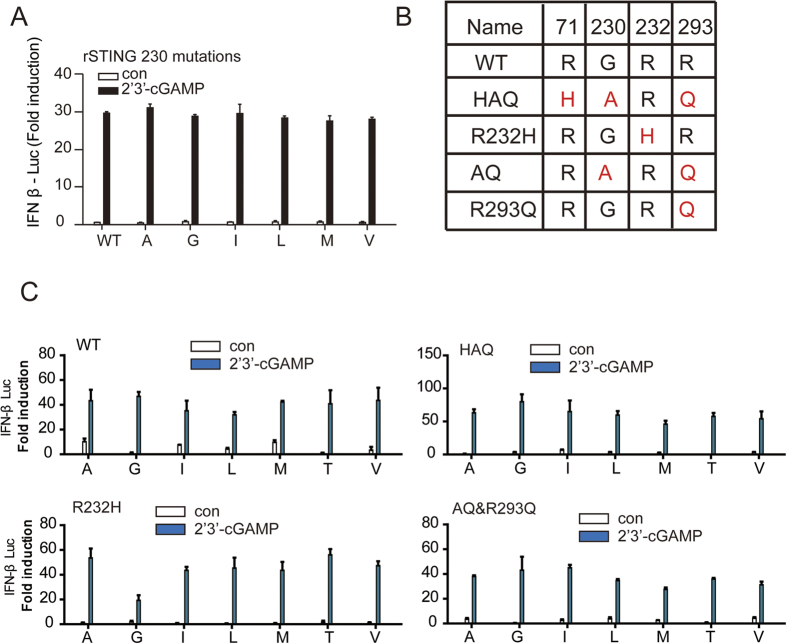
The residue 230 replacement had little effect on cGAMP-induced interferon production. (**A**) Luciferase analysis of the residue 230 mutants in rSTING upon 2′3′-cGAMP stimulation. (**B**) Schematic diagram of hSTING polymorphism. (**C**) Luciferase analysis of the residue 230 mutants in indicated hSTING polymorphism upon stimulated with 2′3′-cGAMP.

Recently, the single nucleotide polymorphisms (SNPs) in hSTING have been identified and analyzed, the genetic variations are found to concentrate in four residues (71, 230, 232 and 293)^16^. The hSTING haplotypes are termed hSTING^WT^, hSTING^HAQ^, hSTING^R232H^, hSTING^AQ^ and hSTING^R293Q^ as explained in Fig. 5b. For residue 230 mutations in the hSTING haplotypes, there were no differences in cGAMP-inducted IFN-β production (Fig. 5c), supporting the idea that only the main chain of residue 230 appears to be important for cGAMP-induced STING signaling. It is worthy to note that we also observed that in rSTING residue 230 mutations affect the drug binding, but do not abolish STING activation (Fig. 5a).

### Functional importance of the hydrophobic interactions between residues 230 and 235

As mentioned above, the DMXAA or CMA binding has been shown to reposition the residues of lid region compared with STING-cGAMP complex, thereby promoting the hydrophobic interactions between residues 230 and 235 (Fig. 3c). Hydrophobic residues in position 230 are expected to strengthen the interactions with residue 235 and thus stabilize the lid region. The glycine or alanine substitution disrupts the hydrophobic interactions between residues 230 and 235, and leads to destabilizing binding agents. Since the bulky side chain of residue 235 confers hydrophobic stabilization, we next investigated whether replacement of residue 235 change the STING signaling after DMXAA or CMA treatment. As shown in Fig. 4d, mSTING^I234A^ and hSTING^I235A^ substitutions significantly decreased the DMXAA or CMA-induced IFN-β induction. Considering that the weak hydrophobic property of T230 in rSTING, rSTING^V235A^ variant showed a slight reduction of IFN-β induction. The data indicates that the hydrophobic interactions between residues 230 and 235 are crucial for anticancer/antiviral compound binding, further demonstrating the functional importance of the lid region. In rSTING-cGAMP structure, the main chain of T230 makes contacts with R238 (Fig. 3c). In addition, no obvious cGAMP-induced signaling change happened after replacement of T230 by other amino acid in rSTING (Fig. 5a), consistent to the structural model where no interaction between the side chain of T230 and the neighboring residues was observed (Fig. 3c). Then we solved the rSTING^T230I^ and rSTING^T230V^ in complex with 2′3′-cGAMP (Table S1), and structural analysis further supports that the main chain of T230 mediates interactions with neighboring residues (Fig. s5). The similar structural features are also found in other available STING-cGAMP structures.

### Replacement of the lid region, especially residue 230, confers rSTING and hSTING sensitivity to CMA and DMXAA.

**Figure 6:**
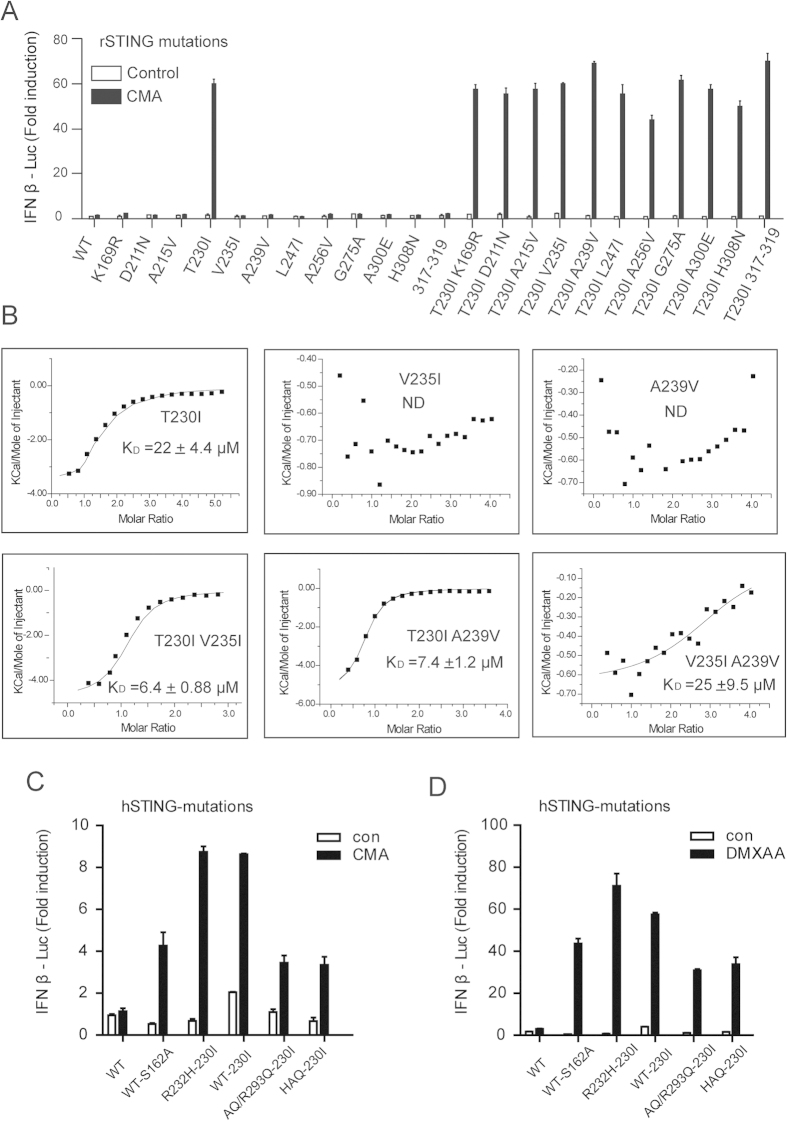
Residue 230 substitutions changed the species selectively for DMXAA or CMA. (**A**) Luciferase analysis of indicated mono- or double- mutants in rSTING upon stimulated with CMA. 317–319 indicates that S317-E319 in rSTING was mutated into the corresponding residues P316-D318 in mSTING. (**B**) CMA binding studies of indicated rSTING mutants by ITC. (**C**) Luciferase analysis of the residue 230 mutants in hSTING upon indicated stimulation.

Despite that the sequence of rSTING is closer to that of mSTING compared with that of hSTING, CMA is still unable to activate rSTING (Fig. 1a). To map the key residues in CMA recognition, we carried out homolog-scanning mutagenesis on rSTING. Strikingly, rSTING^T230I^ variant responded robustly to CMA, but the other single amino acid substitutions were defective in CMA-induced activation (Fig. 6a). As expected, the dual mutation (T230I and any other residue substitution) was also found to activate the CMA-induced IFN-β production. Additionally, rSTING^T230I V235I^ and rSTING^T230I A239V^ variants exhibited moderate increased affinities for CMA compared with rSTING^T230I^ (Fig. 6b). In the light of the observation, we next examined the 230 substitution in hSTING. Similar to that at rSTING^T230I^, hSTING^230I^ variants were able to stimulate the IFN-β production after treatment of CMA (Fig. 6c). Notably, DMXAA also robustly triggered IFN-β production for hSTING^230I^ variants, confirming the pivotal role of position 230 (Fig. 6d). Indeed, replacement of A230 in hSTING by Ile or Leu resulted in detectable binding to DMXAA (Fig. S6). As mentioned above, we have characterized residue 230 as a critical residue involved in drugs binding affinity (mSTING I230 mutations binding of DMXAA or CMA, and rSTING T230 mutations binding of DMXAA) (Fig. 4). The data presented here also indicates position 230 as a versatile regulator of discrimination of anticancer/antiviral compound. Given that rearrangement of residues 235 and 239 in mSTING when binding of CMA (Fig. 3b), we asked whether the dual mutation (V235I and A239V) in rSTING could recognize and bind CMA. Surprisingly, we found that like rSTING^T230I^ variant, the rSTING^V235I A239V^ is sufficient to binding of CMA and shows a considerable affinity (Fig. 6b), suggesting that the key role of lid region in discriminating of anticancer/antiviral agents.

## Discussion

CDNs can function as a second messenger to activate STING-dependent innate immune responses. CDNs were also shown to robustly activate Zebrafish STING (Fig. s7), suggesting the STING-pathway is highly conserved throughout vertebrate evolution. Recent evidences strongly indicate that certain anticancer/antiviral agents including DMXAA and CMA can efficiently target STING–CDNs pathway. However, these compounds exhibit species selectivity and are only found to be effective for mouse STING so far. Thus, the development of STING-targeted drug has been hampered by the lack of an appropriate animal model. In this report we described the rSTING system to study STING activation in response to these compounds. Notably, rSTING responds only weakly to DMXAA and is defective in CMA-induced activation in a very similar manner as that of the hSTING’s response (Fig. 1a–c). The Isothermal titration calorimetry (ITC) data further demonstrate that rSTING and hSTING exhibit similar binding affinity for these sorts of anticancer/antiviral agents (Fig. 1d). Our findings suggest that anticancer/antiviral agents DMXAA and CMA activate rSTING in a similar profile to hSTING and provide a potential animal system for preclinical evaluation of hSTING-targeted drug discovery.

Combined with a functional screening, we identified the key residues for determining anticancer/antiviral agents’ selectivity. Mutation of rSTING T230 to Ile, the corresponding residue in mSTING, restores the CMA-induced activation. The replacement of T230 by hydrophpbic bulky residues such as Ile or Leu, shows significant increased responses to anticancer/antiviral compounds. In contrast, the anticancer/antiviral agents-induced activation and binding affinity are drastically reduced by substitution of T230 in rSTING (or I229 in mSTING) for Gly or Ala. Functionally analysis of hSTING also revealed residue 230 play key roles in ligand-induced signaling.

Compared with STING-cGAMP structure, residue 235 in STING-DMXAA (or STING-CMA) structures undergoes a sharp turn and makes hydrophobic interactions with residue 230, thereby stabilizing the ligand-binding core. We therefore proposed that the corresponding interaction would be absent in A230 (or G230) STING variants. It nicely explains why hydrophobic bulky residues substitution at position 230 show increased binding affinity and higher levels of activation. In this present study, we presented evidence that residue 230 plays crucial role in type I IFN responses to anticancer/antiviral agents. These findings should aid design of STING-targeted drugs. During preparation of our manuscript, Gao et al. reported that the hSTING^G230I^ variant can respond to DMXAA, supported our claim that residue 230 is important for recognition of anticancer/antiviral compounds^35^.

Structural comparisons between STING-cGAMP and STING-agent reveal a rearrangement of the lid region, including the variable residue 230, 235 and 239. Except for the lid region, other parts of STING structure are essentially identical. We first evidenced that CMA directly bind to rSTING^V235I A239V^ mutation. Conformational movements of lid region could also be propagated within the rSTING^V235I A239V^ mutation, and rescuing CMA binding. Our structural and functional analyses highlighted the crucial role of the lid region for the selectivity of anticancer/antiviral agents DMXAA and CMA, although we can not rule out the possibility that other regions may also involved in the agents selectivity.

## Materials and Methods

### Cloning and mutagenesis

The full-length genes of rat STING (rSTING), human STING (hSTING), mouse STING (mSTING) were cloned into p3xFLAG-CMV-14 (Sigma) or pcDNA3.1 (+) vector (Invitrogen) with an HA tag. The C-terminal domain of rSTING (residues 155–341), hSTING (residues 155–341), mSTING (residues 154–340) for ITC measurements, hereafter named rSTING^CTD^, hSTING^CTD^, mSTING^CTD^, were constructed by cloning into pET28a vector between Nde I and Xho I restriction sites, which contains N-terminal TEV protease-cleavable His tag. All point mutations were generated by PCR-based site-directed mutagenesis method with KOD FX as the polymerase. All mutants were confirmed by DNA sequencing.

### Protein expression and purification

The plasimids of pET28a-rSTING^CTD^, hSTING^CTD^, mSTING^CTD^ and their variants were transformed into **Escherichia coli** BL21 (DE3) cells for overexpression. Cells were induced at 18 °C with 0.5 mM isopropyl β-d-1-thiogalactopyranoside for 20 hours, then collected and resuspended in buffer A (20 mM Tris-HCl, pH 8.0, 500 mM NaCl). Cells were subjected to ultrasonic lysis and clarified by centrifugation at 21,500 rpm for 90 minutes. Supernatants were loaded onto a HiTrap HP column (GE Healthcare, USA) equilibrated with buffer A. The contaminants were washed with buffer A containing 100 mM imidazole. The target proteins were eluted with buffer A containing 500 mM imidazole. Eluates were further purified by ion-exchange chromatography (HiTrap Q, GE Healthcare) and eluted with a linear sodium chloride gradient. Further purification steps were conducted through gel filtration (Superdex-200; GE Healthcare) equilibrated with 20 mM Tris–HCl, pH 8.0, 200 mM NaCl. Proteins used for crystallization trials were concentrated to 10 mg ml^−1^.

### Crystallization and data collection

To form complex of rSTING (wt), rSTING (T230I), rSTING (T230V) and 2′3′-cGAMP, the 10 mg ml^−1^ proteins were mixed with 2 mM 2′3′-cGAMP, and incubated on ice for one hour. Crystal screening were performed by sitting-drop vapor-diffusion methods at 18 °C. Crystals of rSTING (apo)^140–341^ were obtained using a reservoir solution composed of 20 mM MES pH 6.5, 200 mM NaCl. Crystals of rSTING-2′3′-cGAMP was obtained using a reservoir solution composed of 1.8 M (NH4)_2_SO_4_, 0.1 M HEPES pH 7.0. The crystals were soaked in a cryoprotectant of crystallization solution containing 20% glycerol before flash-frozen. The data were collected on the Beamline BL17A at the KEK, Photon Factory, Tsukuba, Japan. The diffraction data were mostly integrated and scaled by the program XDS package.

### Structure determination and refinement

The structures of rSTING (apo)^140–341^ and rSTING (wt)-2′3′-cGAMP complex were determined by molecular replacement with PHENIX AutoMR using mSTING (Protein Data Bank ID code: 4LOJ) as the search model. The model was further refined with phenix.refine and the graphics program Coot. The data processing and refinement statistics are summarized in Table 1 and Table S1.

### ITC (Isothermal titration calorimetry) measurements

ITC measurements were performed with an iTC200 instrument (GE Healthcare) at 25 °C. All the proteins and ligands (DMXAA, CMA, 2′3′-cGAMP, 3′3′-cGAMP) were in buffer C (30 mM HEPES, pH7.5, 150 mM NaCl). A typical titration experiment involved 19 injections of ligand (2 mM) solution into the ITC cell containing protein (0.2 mM). The data were fitted to titration curves with Origin v7.0 (MicroCal).

### Cells, Antibodies and General Methods

HEK293T cells, peritoneal macrophages from rat (Sprague-Dawley background) or mouse (C57/BL6 background) were cultured in DMEM supplemented with 10% FBS (Gibco) and antibiotics. THP-1 cells were cultured in RPMI medium supplemented with 10% FBS, L-glutamine, sodium pyruvate.

Anti-Flag (Sigma, F3165), anti-HA (Sigma, H9658), anti-GAPDH (Santa Cruz, sc-25778), anti-p-IRF3 (Epitomics, 2562–1) antibodies were purchased as indicated.

The procedures for SDS–polyacrylamide gel electrophoresis (SDS-PAGE), and Western blotting have been described previously^10^.

### Luciferase Reporter Assay

Reporter assay was performed as previously described^10^. Briefly, HEK293T cells (1×10^5^) were seeded in 24-well plates and transfected with reporter constructs and indicated STING mutation plasmids by standard calcium phosphate precipitation method. After 12 hours, cells were stimulated with medium containing DMXAA (266 μM) or CMA (500 μg ml^−1^) or cells were PFO-permeabilized to deliver cGAMP linkage isomers (5 μM concentration, 30 minutes permeabilization) and incubated for an additional 12 hours, followed by luciferase reporter assay. PFO assay was previously described^36^.

### Isolation of Peritoneal Macrophages

Isolation of macrophages was performed as previously described^37^. Briefly, Peritoneal macrophages were harvested from rats or mice 4–5 days after thioglycollate (BD, Sparks, MD) injection (15–20 mL for rat and 1.5–2 mL for mice), and cultured in DMEM supplemented with 10% FBS. Cells were plated into 6-well plates and stimulated with DMXAA (266 μM) or CMA (500 μg ml^−1^) or cells were PFO-permeabilized to deliver cGAMP linkage isomers (5 μM concentration, 30 minutes permeabilization) 12 hours later, cytokine production was analyzed by RT-PCR. All experimental protocols involving rats and mice were approved by the Laboratory Animal Center of Peking University. The animals were treated humanely according to the institutional animal care guidelines.

### RT-PCR

Total RNA was extracted with Trizol reagent (Invitrogen) and reversed-transcribed with Reverse Transcription System (Promega). Indicated gene induction was analyzed by RT-PCR for 25–30 cycles at 94 °C for 30 seconds, 58 °C for 30 seconds and 72 °C for 30–50 seconds. Primers for RT-PCR are shown in the following table 2.

### Statistical Analysis

We performed statistical analysis by Student’s t-test. We considered P < 0.05 to be statistically significant.

### Ethics Statements

Mice and Rats were kept at the Laboratory Animal Center of Peking University. All animal studies were conducted at the AAALAC approved Animal Facility in the Laboratory Animal Center of Peking University. Experiments were carried out in conformity with protocols (*LSC-JiangZF-2* entitled “Innate immunity and related signaling transduction”) approved by Peking University Laboratory Animal Center (Beijing, China), in accordance with the National Institute of Health Guide for Care and Use of Laboratory Animals. All efforts were made to minimize the animal’s suffering.

## Additional Information

**How to cite this article**: Zhang, H. *et al.* Rat and human STINGs profile similarly towards anticancer/antiviral compounds. *Sci. Rep.* **5**, 18035; doi: 10.1038/srep18035 (2015).

## Supplementary Material

Supplementary Information

## Figures and Tables

**Table 1:** Data Collection and Refinement Statistics for rSTING alone and rSTING- 2′3′-cGAMP complex.

Data	rSTING_140–341_	rSTING_155–341_+2′3′cGAMP
Space group	C2	P4_1_2_1_2
Unit Cell: a, b, c (Å)	138.9 75.5 87.8	77.2 77.2 152.1
α, β, γ (°)	90 115.3 90	90 90 90
Resolution (Å)	(2.0–1.9)	(1.6–1.55)
R_merge_ (%)^b^	7.8(52.5)	5.2(57.2)
Mean **I**/sigma(**I**)	14.21(2.94)	20.1(3.45)
Completeness (%)	96.9(93.6)	99.8(100)
Number of measured reflections	193088(30085)	480508(43063)
Number of unique reflections	63573(9860)	67310(6032)
Refinement		
Resolution range(Å)	50–1.9	50–1.55
R_work_ (%)/ R_free_ (%)	18.12/23.04	18.70/21.21
No.atoms		
protein	6569	3020
Ligand		45
Water	600	410
B factors		
Protein	24.00	20.0
Ligand	28.90	19.50
Water	29.10	29.70
R.m.s.deviations		
Bond lengths(Å)	0.008	0.012
Bond angles(°)	1.20	1.65
Ramachandran favored (%)	96	98
Ramachandran outlines (%)	0.49	0

**Table 2:** RT-PCR primers

Species	Genes	Forward Primer	Reverse Primer	Product Size
Homo	*Ifnb*	CTAACTGCAACCTTTCGAAGC	CTAGTGTCCTTTCATATGCAG	746bp
	*Cxcl10*	GGAACCTCCAGTCTCAGCACC	GGCAGTGGAAGTCCATGAAGTAA	756bp
	*Il6*	CTTCGGTCCAGTTGCCTTCT	GTGCCCATGCTACATTTGCC	625bp
	*Hprt*	CTGGCGTCGTGATTAGTGATG	TATCCAACACTTCGTGGGGTC	505bp
Rat	*Ifnb*	CAACCTCAGCTACAGGACGG	GACCACCATCCAGGCATAGC	346bp
	*Cxcl10*	CGCATGTTGAGATCATTGCC	CTGCCTGAGGGAAGATTCGG	365bp
	*Il6*	GTTTCTCTCCGCAAGAGACTTC	ACGGAACTCCAGAAGACCAGAG	356bp
	*Hprt*	CAGCGTCGTGATTAGTGATG	CTTCAACAATCAAGACGTTC	386bp
Mice	*Ifnb*	TCCAGCTCCAAGAAAGGACG	CTACCAGTCCCAGAGTCCGC	551bp
	*Cxcl10*	ATGACGGGCCAGTGAGAATG	CCCTTGGGAAGATGGTGGTT	648bp
	*Il6*	TCCAGTTGCCTTCTTGGGAC	AACGCACTAGGTTTGCCGAG	613bp
	*Hprt*	GGACAGGACTGAAAGACTTGCTCG	TCCAACAAAGTCTGGCCTGTATCC	412bp
