# Supplementary material for: Rat and human STINGs profile similarly towards anticancer/antiviral compounds
Source: Sci Rep. 2015 Dec 16;5:18035. doi: 10.1038/srep18035 (PMC4680857; doi:10.1038/srep18035)
Supplement: Supplementary Information [file srep18035-s1.doc]

**Supplementary information**

**Rat and human STINGs profile similarly towards anticancer/antiviral compounds**

Heng Zhang, Ming-Jie Han, Jian-Li Tao, Zhao-Yang Ye, Xiao-Xia Du, Ming-Jing Deng, Xiao-Yan Zhang, Lan-Fen Li, Zheng-Fan Jiang and Xiao-Dong Su

**Supplementary Table**

**Table S1** Data Collection and Refinement Statistics for rSTING_155-341_^T230I^ and rSTING_155-341_^T230V^ in complex with 2’3’-cGAMP

| Data | rSTING_155-341_^T230I^ | rSTING_155-341_^T230V^ |
| --- | --- | --- |
| Space group | P 4_1_ | P 4_1_ |
| Unit Cell:  a, b, c (Å) | 77.2 77.2 149.9 | 77.2 77.2 150.6 |
| α, β, γ (°) | 90 90 90 | 90 90 90 |
| Resolution (Å) | 38.6 -1.80 (1.86 -1.80) | 38.6-1.8(1.90-1.80) |
| R_merge_ (%)b | 8.5 (59.0) | 5.4 (52.6) |
| Mean I/sigma(I) | 26.13 (5.12) | 12.15 (1.74) |
| Completeness (%) | 99.9 (99.5) | 99.8 (97.6) |
| Redundancy | 1.36 (5.62) | 2.66 (2.61) |
| **Refinement** |  |  |
| Resolution range(Å) | 38.6 -1.80 (1.86 -1.80) | 38.6-1.8 (1.9-1.8) |
| R_work_ (%)/ R_free_ (%) | 19.5/21.9 | 19.2/21.7 |
| Average B-factors | 29.80 | 24.40 |
| R.m.s.deviations |  |  |
| Bond lengths(Å) | 0.013 | 0.010 |
| Bond angles(°) | 1.44 | 1.30 |
| Ramachandran plots  Ramachandran favored（%） Ramachandran disallowed（%） | 97  0.41 | 97  0.41 |

R_merge_ = ∑|Ii -< Ii >|/∑|Ii|, where Ii is the scaled intensity of the ith measurement, and < Ii > is the mean intensity for that reflection.

R_work_ = ∑||Fobs|-|Fcalc||/∑|Fobs| where Fcalc and Fobs are the calculated and observed structure factor amplitudes, respectively

R_free_=as for Rwork, but for 5.0% of the total reflections chosen at random and omitted from refinement.

**Supplementary Figures**

**
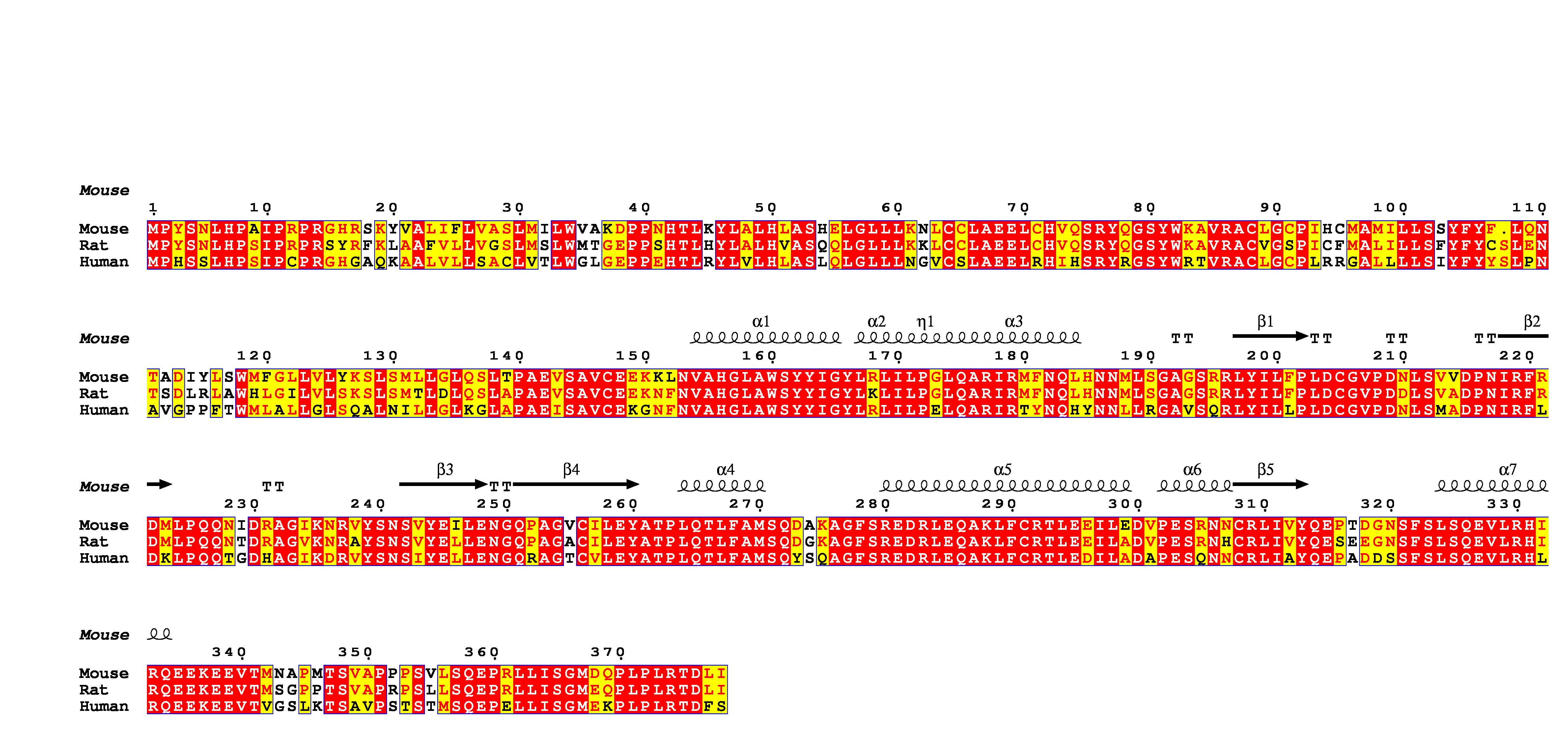
**

Firgure S1, Sequence alignments of rSTING, mSTING and hSTING.


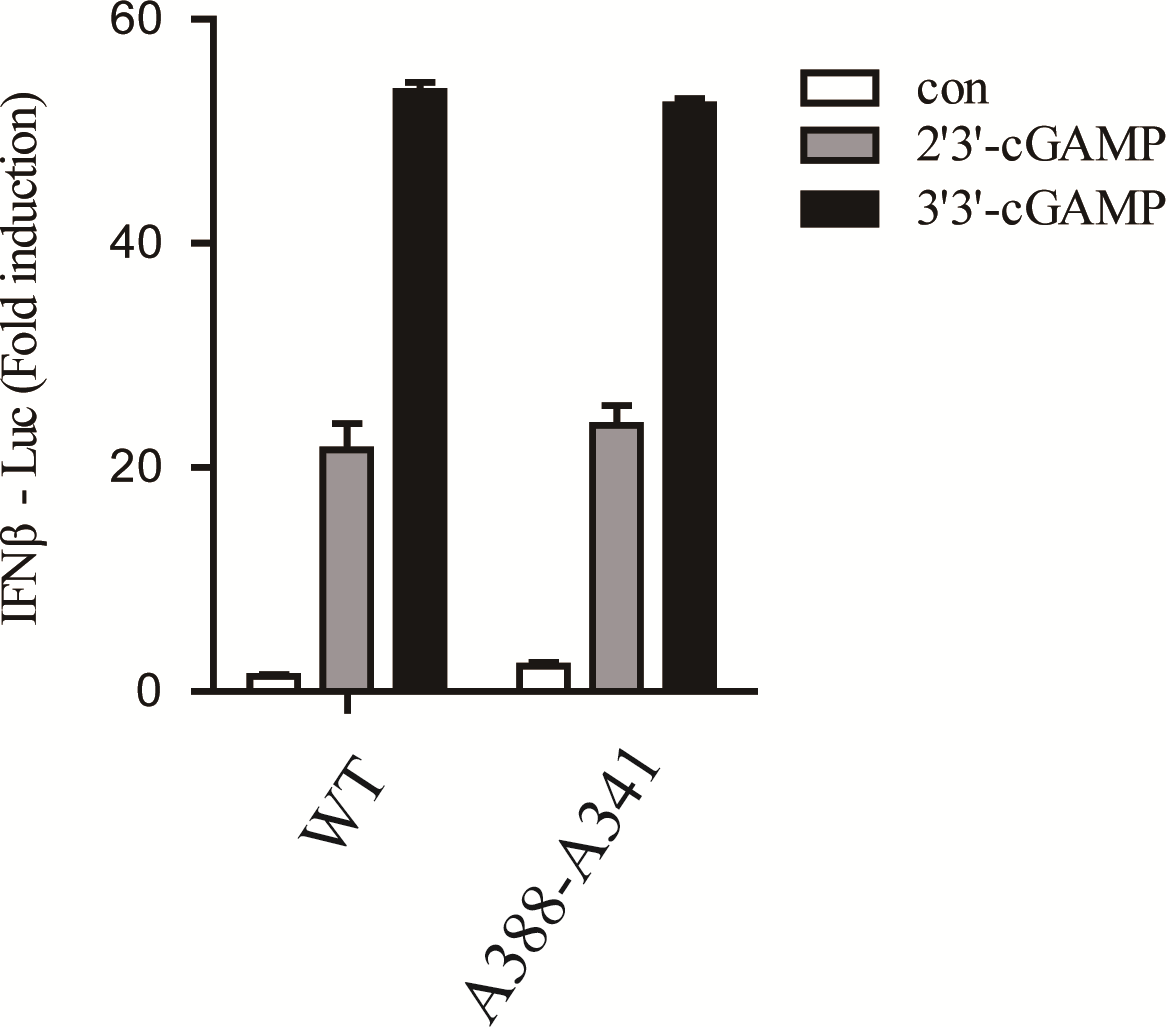


Figure S2, A388-A341 mutations in rSTING showed little effect on cGAMP-induced

signaling. Luciferase analysis of WT or mutants in rSTING upon stimulated with 2’3’-cGAMP or 3’3’-cGAMP.


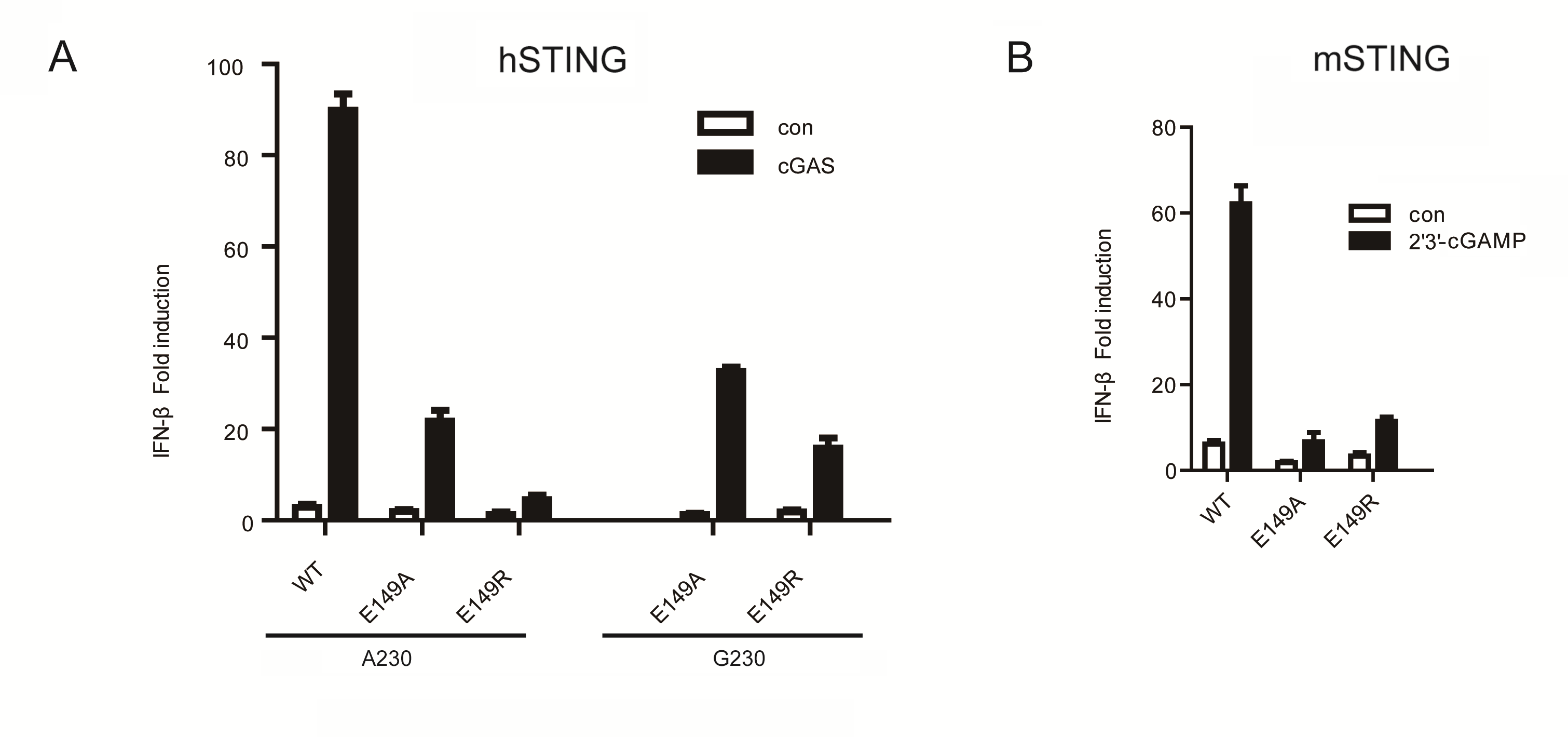


Figure S3, E149A or E149R mutations in mSTING/hSTING remarkably decreased the type I interferon signaling.

(A) 293T cells were transiently transfected with 5ng indicated hSTING mutants and 25ng cGAS expression plasmids, along with 50ng IFN β -Luc reporter plasmids. 24 hr after transfection, a luciferase assay was then performed.

(B) Luciferase analysis of WT or mutants in mSTING upon stimulated with 2’3’-cGAMP.


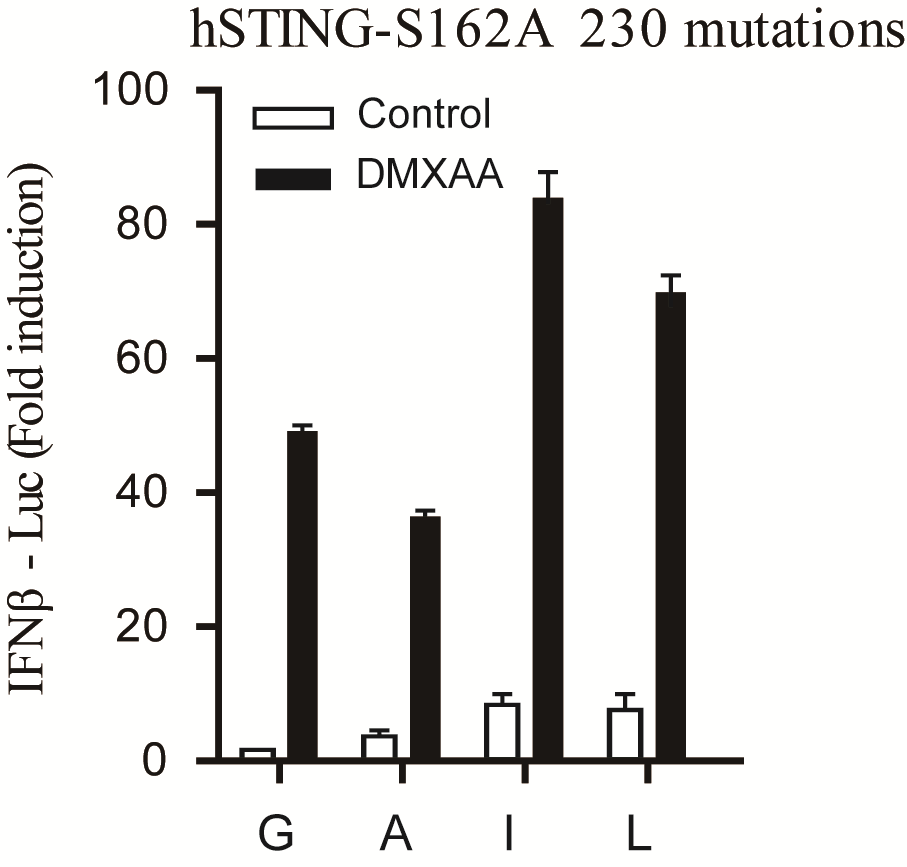


Figure S4, Luciferase analysis of 230 substitutions in hSTING^S162A^ upon stimulated with DMXAA.


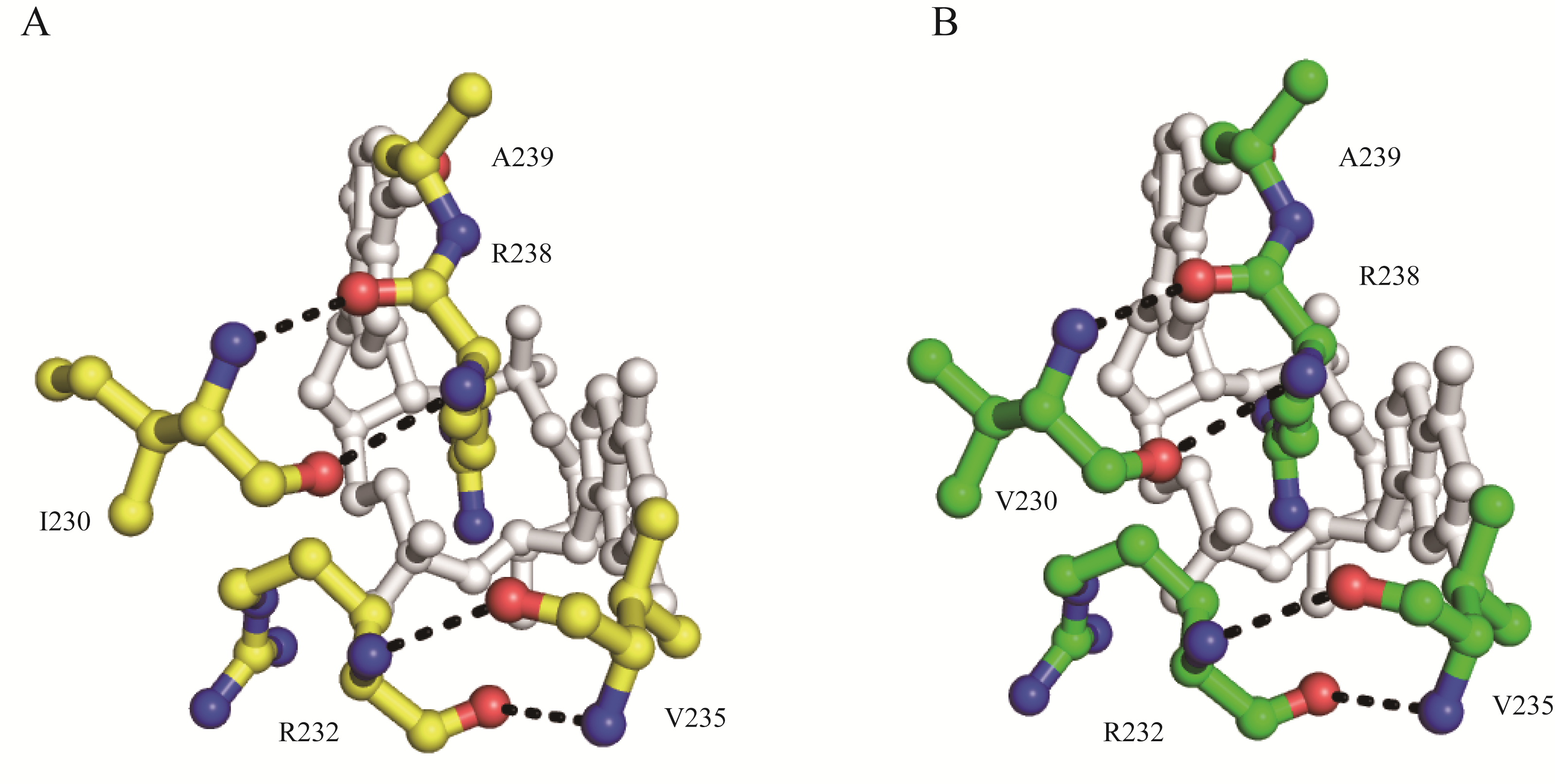


Figure S5, A close-up view of residue 230 variants involved in interactions.


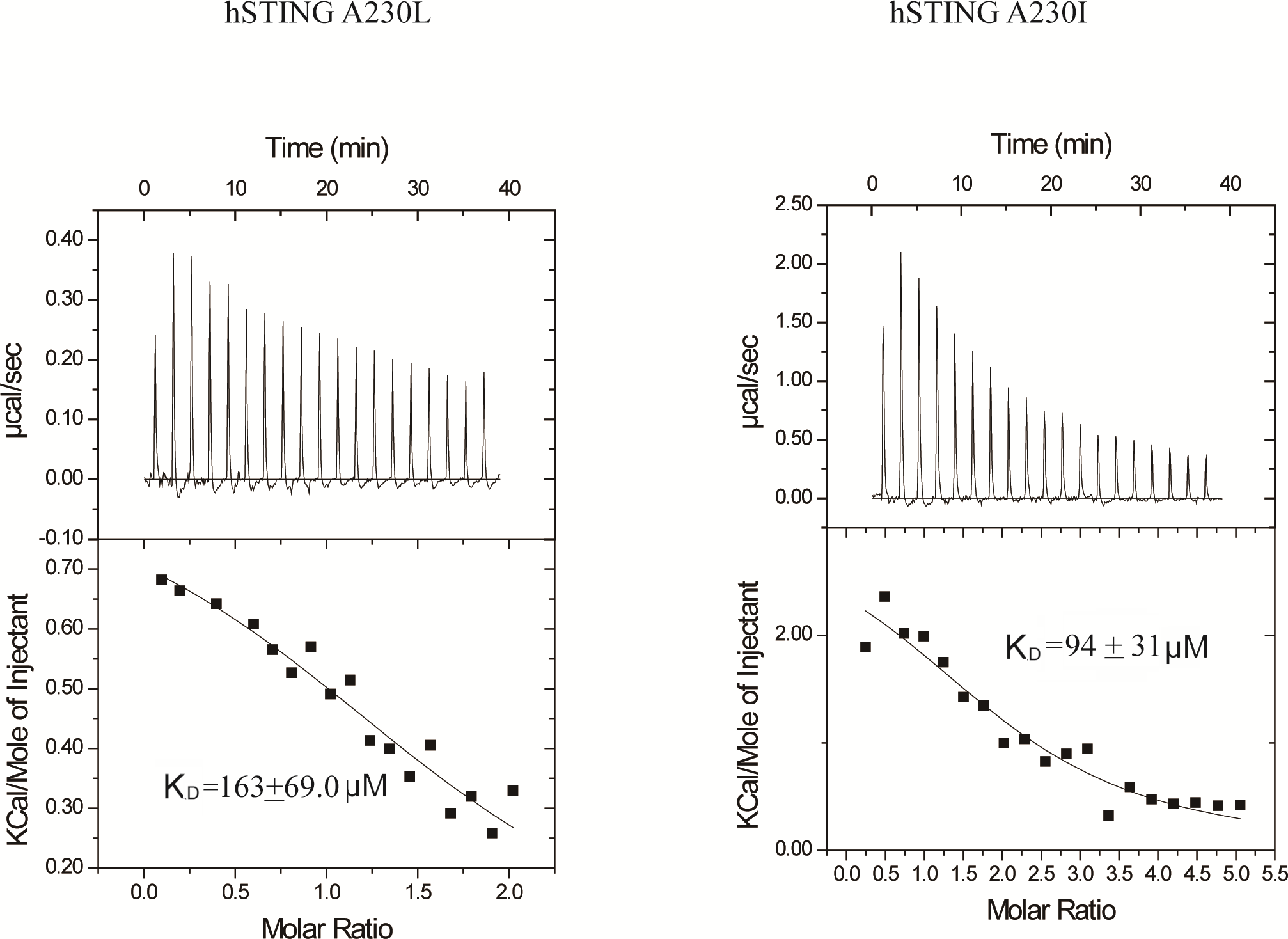


Figure S6, DMXAA binding studies of indicated hSTING mutants by ITC.


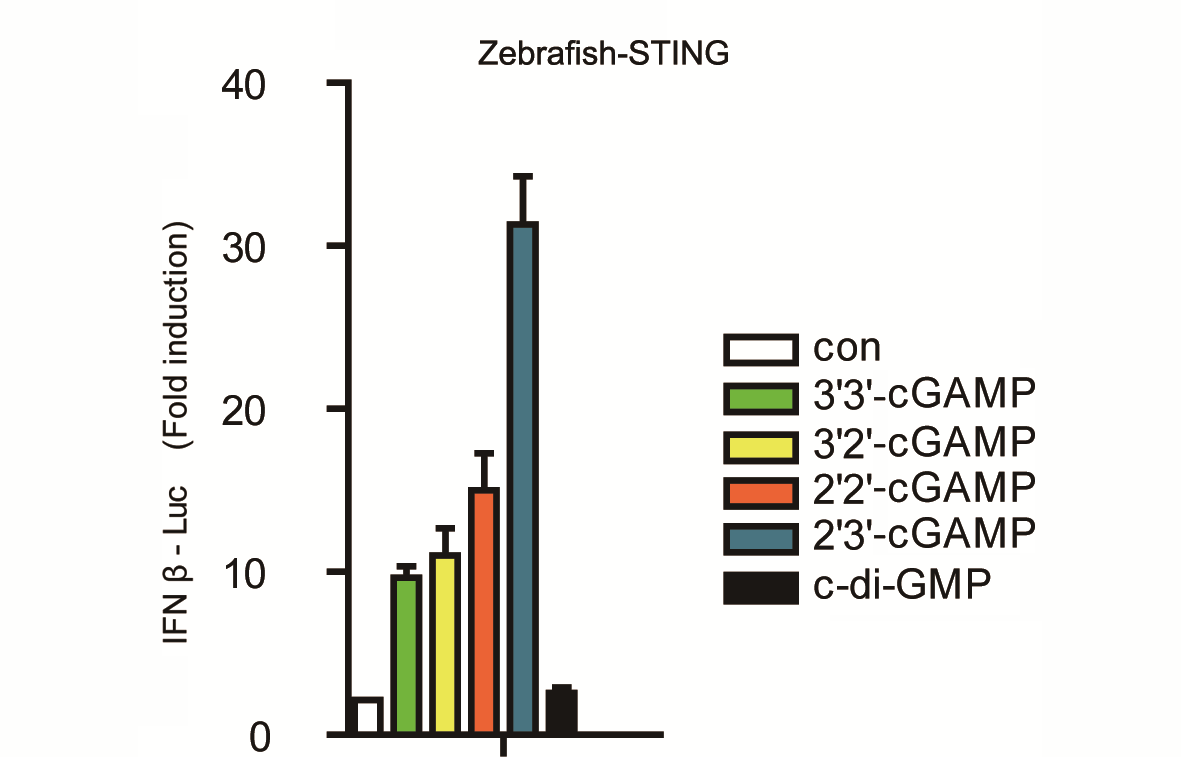


Figure S7, Luciferase assay of Zebrafish STING in response to CDNs.


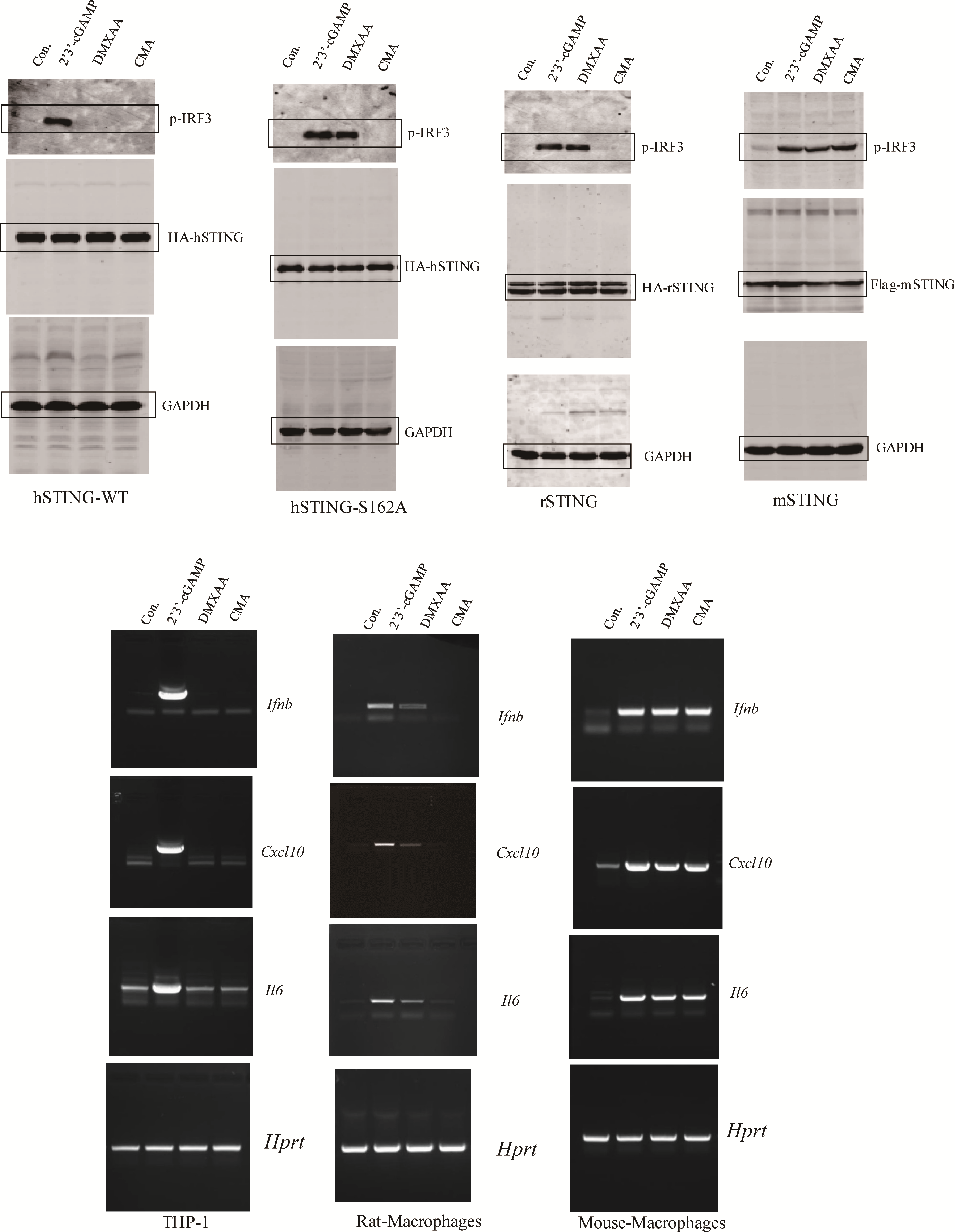


Figure S8, Full-length immunoblots and RT-PCR.

Full immunoblots and RT-PCR corresponding to the cropped versions shown in Fig. 1b and Fig.1C are shown.


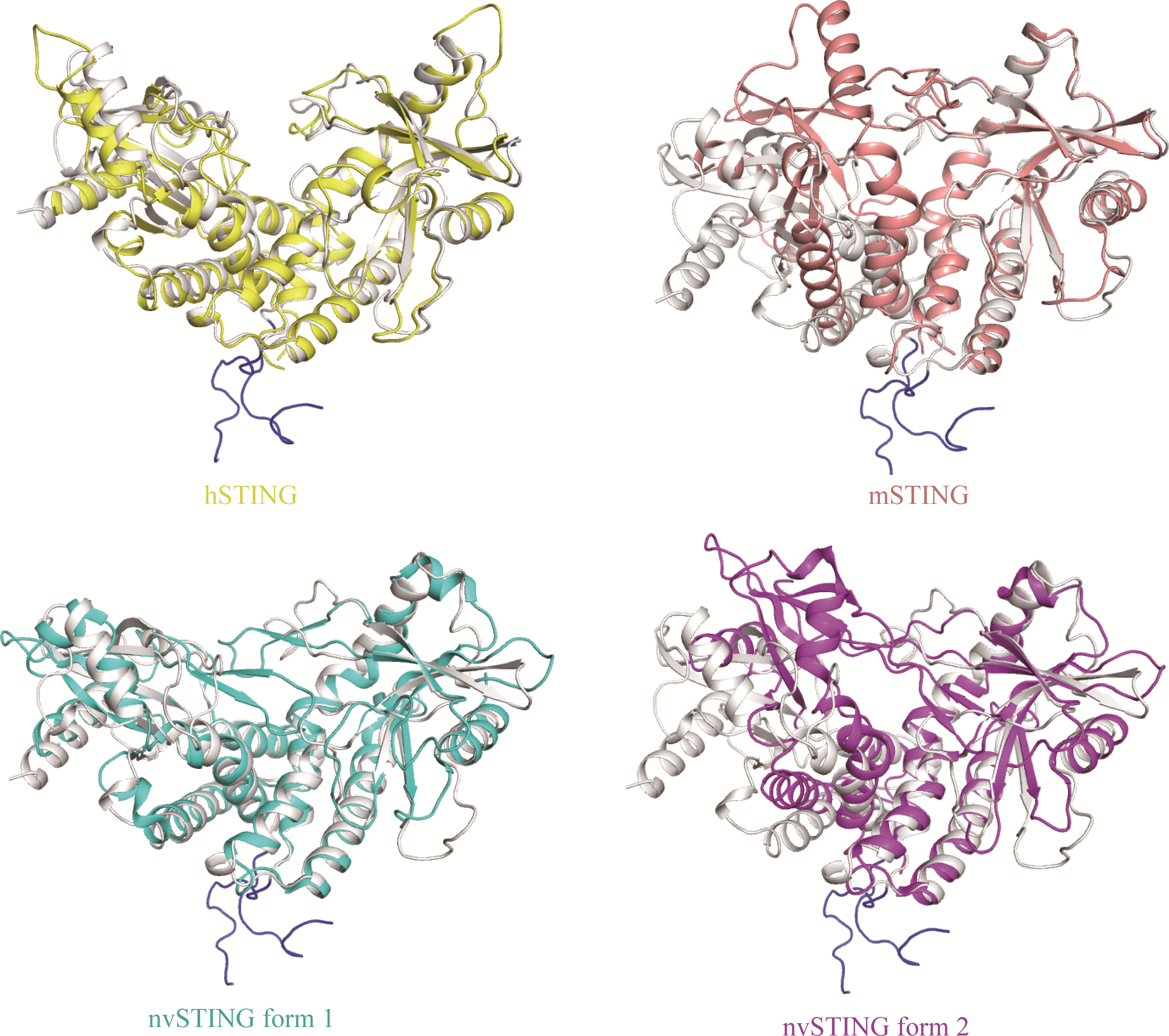


Figure S9,

Structural comparisons of native rSTING (grey) with native hSTING (yellow, PDB: 4F5E), native mSTING (orange, PDB: 4KC0), native nvSTING form 1(cyan, PDB: 5CFO) and native nvSTING form 2 (magenta, PDB: 5CFR).


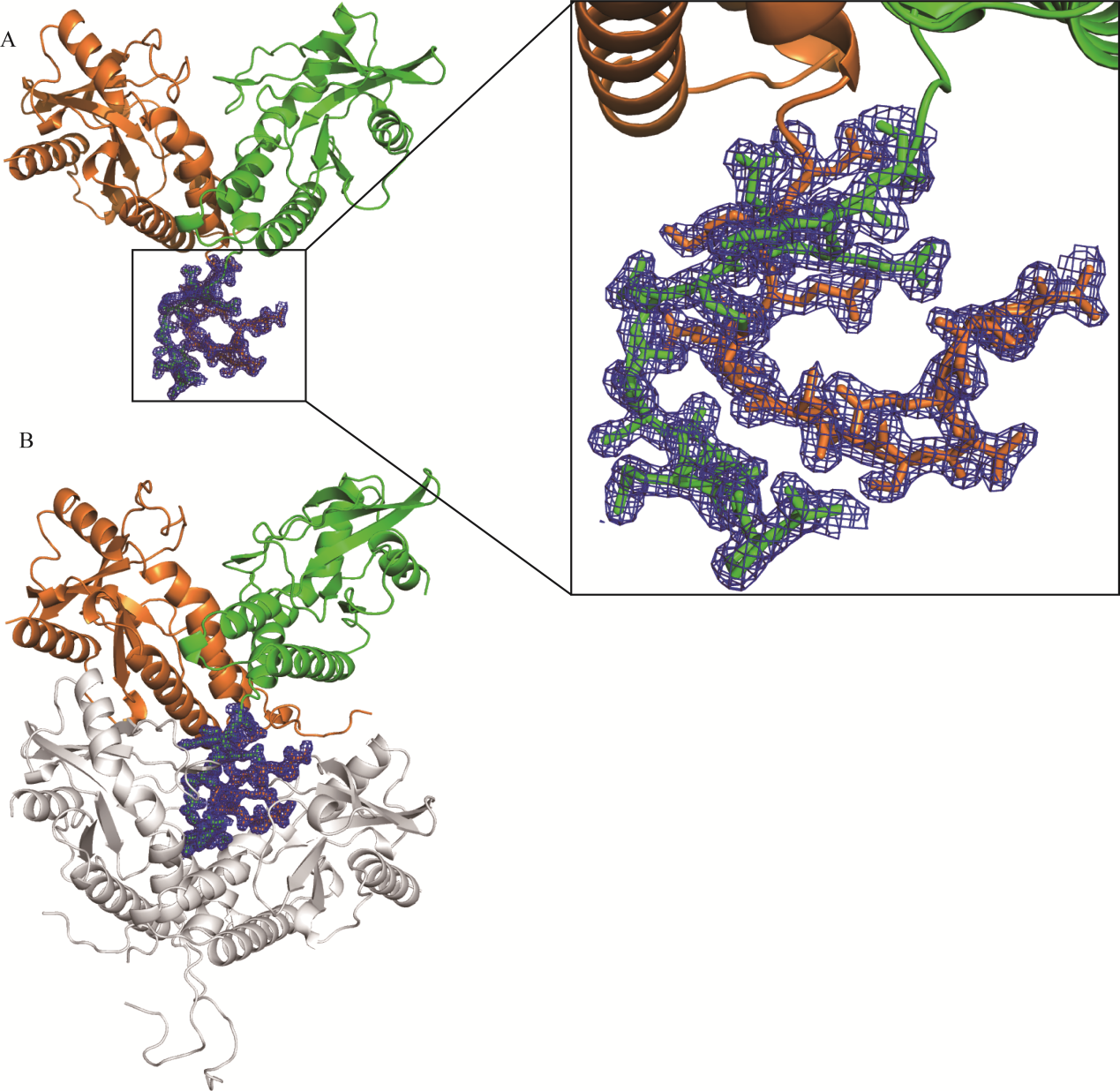


Figure S10,

Stereo view of extended loop (A140-N152).

1. The 2*Fo-Fc* electron-density maps contoured at 1.2 σ for the extended loop. The chain of rSTING is highlighted in a orange or green color. The left panel shows a close-up presentation of the extended loop.
2. The extended loop makes interactions with the neighboring molecule in the crystal lattice, suggesting the structural dynamic of the native STING.
